# Microbial prodigiosin shows broad-spectrum bioactivity confirmed by experimental and computational analyses

**DOI:** 10.3389/fmicb.2025.1676959

**Published:** 2025-09-24

**Authors:** Muhammad Rafiq, Noor ul Huda, Noor Hassan, Hazrat Ali, Abdul Tawab, Rizwan Bashir, Naveed Iqbal, Zara Rafaque, Faisal Ahmad, Yanyan Wang, Waqar Rauf, Anam Saqib, Iqra Jawad, Yingqian Kang

**Affiliations:** ^1^Guizhou Key Laboratory of Microbiome and Infectious Disease Prevention and Control, Key Laboratory of Environmental Pollution Monitoring and Disease Control, Ministry of Education of Guizhou, School of Basic Medical Sciences, Joint Laboratory of Shanghai Dongli One Health Research Institute Co., Ltd., Guizhou Medical University One Health Research Institute, Guiyang, China; ^2^Department of Microbiology, Faculty of Life Sciences and Informatics, Balochistan University of IT, Engineering and Management Sciences, Quetta, Pakistan; ^3^National Institute for Biotechnology and Genetic Engineering–College (NIBGE-C), Pakistan Institute of Engineering and Applied Sciences (PIEAS), Faisalabad, Pakistan; ^4^Department of Biotechnology, Faculty of Life Sciences and Informatics, Balochistan University of IT, Engineering and Management Sciences, Quetta, Pakistan; ^5^The Department of Paediatrics and Child Health, Aga Khan University, Karachi, Pakistan; ^6^Department of Microbiology, Hazara University, Mansehra, Pakistan; ^7^National Institute of Health, Islamabad, Pakistan; ^8^DAI Fleming Fund Country Grant, Islamabad, Pakistan

**Keywords:** antibacterial, antifungal, antioxidant, prodigiosin, secondary metabolite, *Serratia marcescens*

## Abstract

The growing demand for natural bioactive compounds highlights the need for antimicrobial and antioxidative metabolites derived from microbial sources. Among them, prodigiosin, a red pigment from *Serratia marcescens*, displays potent antioxidant and antimicrobial properties. However, optimizing its production and understanding molecular interactions remain challenging. In this study, we identified an optimized process for enhanced yield using peptone meat extract (PM) media at an incubation temperature of 30 °C, which notably outperformed other tested conditions and media. The purified red pigment was further characterized by column and thin-layer chromatography, UV–visible spectrophotometry, Fourier Transform Infrared (FT-IR) spectroscopy, and Mass Spectrometry (ESI-MS/MS). The pigment demonstrated an R*_f_* value of 0.93 through column chromatography and TLC. The structural characteristics were established using UV–Vis (λ_max_ 536 nm), FT-IR, and ESI-MS/MS (m/z 324.3 amu), consistent with the prodiginine family. The characterized and purified prodigiosin showed excellent antibacterial activity against Gram-negative bacteria *Escherichia coli* (28.2 ± 0.57 mm) and Gram-positive bacteria *Bacillus subtilis* (23.58 ± 0.6 mm), together with antifungal activity against *Fusarium oxysporum* and *Aspergillus niger*. Antioxidant analysis showed a dose-dependent radical-scavenging activity of up to 37.5% at 1000 μg/mL. To understand the mechanistic pathways, molecular docking revealed high binding affinities of the produced metabolite with key target sites as FKS1 (−7.2 kcal/mol) for antifungal inhibition, FabH (−7.3 kcal/mol) against antibacterial inhibition, and Keap1 (−8.3 kcal/mol) for antioxidant activity. Our findings not only feature prodigiosin’s broad-spectrum bioactivity but also offer its interaction with molecular targets, providing the basis for developing this metabolite as a natural therapeutic agent in multiple industrial applications, including pharmaceuticals and agriculture.

## 1 Introduction

Microorganisms harbor nature’s vault, anticipating strategic development to offer promising high-value-added products for humans. The genus *Serratia* presented various potential avenues for the biotechnological production of extracellular products such as pigments, biosurfactants, exoenzymes, and nucleases (Grimont and Grimont, 2006). *Serratia marcescens*, belonging to the Enterobacteriaceae family, is a Gram-negative, rod-shaped, flagellated saprophytic bacterium (Hejazi and Falkiner, 1997). The facultative anaerobe has been recovered from various habitats, encompassing air, water, soil, animals, and plants (Grimont and Grimont, 1978; Mahlen, 2011). Notably, the history of pathogenic bacterium, causing nosocomial infections (Mahlen, 2011), has attracted the attention of scientific research because of its unique chromogenic properties and bioactivities of microbially produced pigments. Thus, *S. marcescens* was predominantly recognized as a prolific producer of water-insoluble and non-diffusible red pigments called prodigiosin.

Prodigiosin (C_20_H_25_N_3_O), a red tripyrrole pigment, belongs to the bioactive colored family of molecules known as prodiginines. Prodigiosin was marked as a pyrryldipyrrylmethane skeleton comprising a 4-methoxy-2,2′-bipyrrole unit connected to a monopyrrole unit through a methylene bridge (Hearn et al., 1970; Williamson et al., 2006). The biosynthesis of prodigiosin in *Serratia* species is regulated through prodigiosin biosynthetic cluster genes *A–N* (Williamson et al., 2006), quorum sensing (Sakuraoka et al., 2019), and multiple transcription factors (Gristwood et al., 2011; Tanikawa et al., 2006; Zhao et al., 2023). Moreover, various physiological and biochemical factors, such as carbon and nitrogen sources, incubation period, temperature, aeration, and pH, influence the production of pigments, even within the same genus or species (Bhagwat and Padalia, 2020). Emerging evidence proves that *S. marcescens* is a model organism for the production of multifunctional alkaloid molecules (Dos Santos et al., 2021; Tran et al., 2021).

The high-value compound prodigiosin showed exceptional biological activities and pigmentation properties. It is mainly recognized for its significant antibacterial activity against Gram-negative bacteria, such as *Escherichia coli* and *Pseudomonas aeruginosa* (Yip et al., 2021), and Gram-positive bacteria, such as *Bacillus subtilis* (Danevčič et al., 2016) and *Staphylococcus aureus* (Lim et al., 2022). Furthermore, prodigiosin exhibited antifungal activity against a limited range of fungal pathogens, including *Helminthosporium sativum*, *Rhizoctonia solani*, *Fusarium oxysporum*, and *Candida albicans* (Parani and Saha, 2008). Additionally, the non-toxic and biodegradable properties of pigments exhibited a promising and wide array of potential applications in various sectors of agriculture, pharmaceuticals, food processing, and textile industries (Numan et al., 2018).

In this context, a critical aspect lies in the search for efficient production media, providing conducive conditions for the pigment production of prodigiosin. Different nutrient media significantly affect pigment yield and, ultimately, large-scale production processes for numerous applications (de Araújo et al., 2010; Gulani et al., 2012). The current research focuses on offering valuable insights into the production of biologically active red pigments from *S. marcescens* in various production media. Subsequently, by the structural elucidation of the red pigment, analytical techniques were used for further investigation.

The current study also assessed the biological activity of the purified pigments against both Gram-negative and Gram-positive bacteria and fungal pathogens. In addition, the antioxidant activity of red pigment was investigated for multifaceted applications in therapeutics and other fields. The present study may prompt researchers to evaluate the biological potential of various secondary metabolites as pigments from diverse microbial sources in the future.

## 2 Materials and methods

### 2.1 Recovery of *S. marcescens*

A previously isolated and identified *S. marcescens*, preserved in glycerol stock at −20 °C, was used in the current study (Hassan et al., 2019). The strain was revived on Luria-Bertani (LB) agar media with the following compositions (gL^−1^): (tryptone: 10, yeast extract: 5, sodium chloride: 10, agar: 25). Colony formation and pigmentation of *S. marcescens* were observed on plates incubated for 48 h at 30 °C. The pigment-producing strain was subcultured on LB agar to evaluate the viability of the respective strain.

### 2.2 Inoculum development

For seed culture preparation, 100 mL of seed medium was prepared in a 250 mL Erlenmeyer flask with the following composition (gL^−1^): (peptone: 10, yeast extract: 5, sodium chloride: 3, and potassium chloride: 2). The pH of the media was adjusted to 7.0 with 1 N HCl or 1 N NaOH before autoclaving. A loopful of the revived culture of *S. marcescens* from the LB plate was inoculated into the prepared seed medium, followed by incubation for 24 h at 30 °C at 180 rpm in an orbital shaker incubator.

### 2.3 Production of prodigiosin

Different production media were used for pigment production from *S. marcescens* through submerged fermentation. A 200 mL of each production media was prepared in 500 mL Erlenmeyer flask with compositions as follows (gL^−1^); Nutrient Broth (NB); (peptone: 5, yeast extract: 5, sodium chloride: 3); Luria- Bertani (LB) broth; (tryptone: 10, yeast extract: 5, sodium chloride: 10); Kings B medium; (glycerol: 1%, peptone: 20, dipotassium phosphate: 1.5, magnesium sulphate: 1.5); Peptone-meat extract (PM) broth; (peptone: 5, meat extract: 3); Peptone-glycerol (PG) broth; (peptone: 5, glycerol: 1%); Glucose-Yeast-Malt extract (GYM) broth (glucose: 4, yeast extract: 4, malt extract: 10, calcium carbonate: 2); Minimal media (MM) broth; (glycerol: 0.3%, tween-80: 1.8%, peptone: 15, magnesium sulphate: 2, potassium chloride: 3, sodium chloride: 2). The pH of the media was adjusted to 7.0 with 1 N HCl or 1 N NaOH before autoclaving. Subsequently, a flask containing each production medium was inoculated with 4% (v/v) inoculum from the seed medium and incubated for 72 h at 30 °C and 37 °C at 180 rpm in an orbital shaker incubator for pigment production.

Preliminary screening of the production media was carried out for the selection of optimal media for pigment production. The production media exhibiting the highest efficacy were scaled up to 1,500 mL for the production of red pigment for further analysis.

### 2.4 Preliminary identification of pigment

The pigment was initially identified through a presumptive color test for prodigiosin, as described in a previous study (Gerber and Lechevalier, 1976). Fermentation broth (10 mL) was mixed with absolute methanol and centrifuged at 10,000 rpm for 15 min. The cell-free supernatant was recovered, and the clear solution was divided into two test tubes. One of the test tubes was alkalinized with a concentrated sodium hydroxide (NaOH) solution, and the other was acidified with a concentrated hydrochloric acid (HCl) solution. A color change in the solution was observed to validate the presence of prodigiosin.

### 2.5 Extraction and purification of prodigiosin

Prodigiosin extraction was conducted using a modified version of the protocol described in a previous study (Liu et al., 2021). After a 72-h incubation period, the production media exhibiting visible red pigmentation were subjected to extraction with absolute methanol. The fermentation broth was mixed with absolute methanol at a ratio of 4:1 (v/v) and incubated at 30 °C at 180 rpm in a shaker incubator for 30 min. Subsequently, the mixture was vortexed vigorously in a vortex mixer to promote pigment extraction, followed by centrifugation at 10,000 rpm at 4 °C for 20 min until the pellet became colorless. The resulting supernatant-containing pigment was recovered and concentrated using a rotary vacuum evaporator (Hei-VAP Core; Heidolph, Germany) at 45 °C, to obtain the crude prodigiosin. The crude pigment extract was freeze-dried and stored at 4 °C for further use.

Following this, powdered pigment was dissolved in a minimum volume of methanol and purified by silica gel column chromatography. The silica gel 60 with a mesh size of 70–230 ASTM (Scharlau) was used as an adsorbent for the separation of non-colored impurities from a pigment. The crude pigment was loaded onto a silica gel column, balanced with a solvent system comprising chloroform and methanol (9:1, v/v), and run through the column at a flow rate of 1 mL min ^−1^. The eluted fractions of red color were collected and concentrated under vacuum using a rotary evaporator at 45 °C. The red pigment was further purified by thin-layer chromatography (TLC) on a silica gel 60 F_254_ TLC plate (Sigma-Aldrich) with dimensions of 20 × 20 cm. A solvent mixture of chloroform and methanol (9:1, v/v) was used as the mobile phase. The separated spots and solvent front were carefully marked for the calculation of the Retention Factor (*R_f_*) value. The *R_f_* value of the separated compound was calculated and compared with the literature-reported value for identification of the compound.


$$\begin{array}{l} R_{f}\;\text{value}=\text{Distance travelled}\;\mathrm{by}\;\text{the component}/\\ \;\;\;\;\;\;\;\;\;\;\;\;\;\;\;\;\;\;\;\;\;\;\;\;\;\;\text{Distance travelled}\;\mathrm{by}\;\text{the solvent} \end{array}$$

### 2.6 Characterization of prodigiosin

To characterize the pigment produced by *S. marcescens,* it was subjected to various analytical techniques, as described below.

#### 2.6.1 UV–vis spectrophotometric analysis

To determine the maximum absorbance (λ_max_), the purified red pigment was subjected to UV–vis spectrophotometry. For this purpose, the pigment was dissolved in methanol and scanned in the wavelength range 500–550 nm using a UV double-beam spectrophotometer (Dynamica Halo DB-20, Austria), with absolute methanol serving as a blank, and the spectral data were recorded.

#### 2.6.2 Fourier transform infrared (FT-IR) spectroscopic analysis

FTIR analysis was carried out to determine the functional groups present in the molecules of the red pigment in the spectral range 4,000–400 cm^−1^. The characteristic peaks corresponding to the intricate functional groups of the prodigiosin compound were identified and compared with reference spectra reported in the literature.

#### 2.6.3 ESI-MS/MS of red pigment

**Figure 1:**
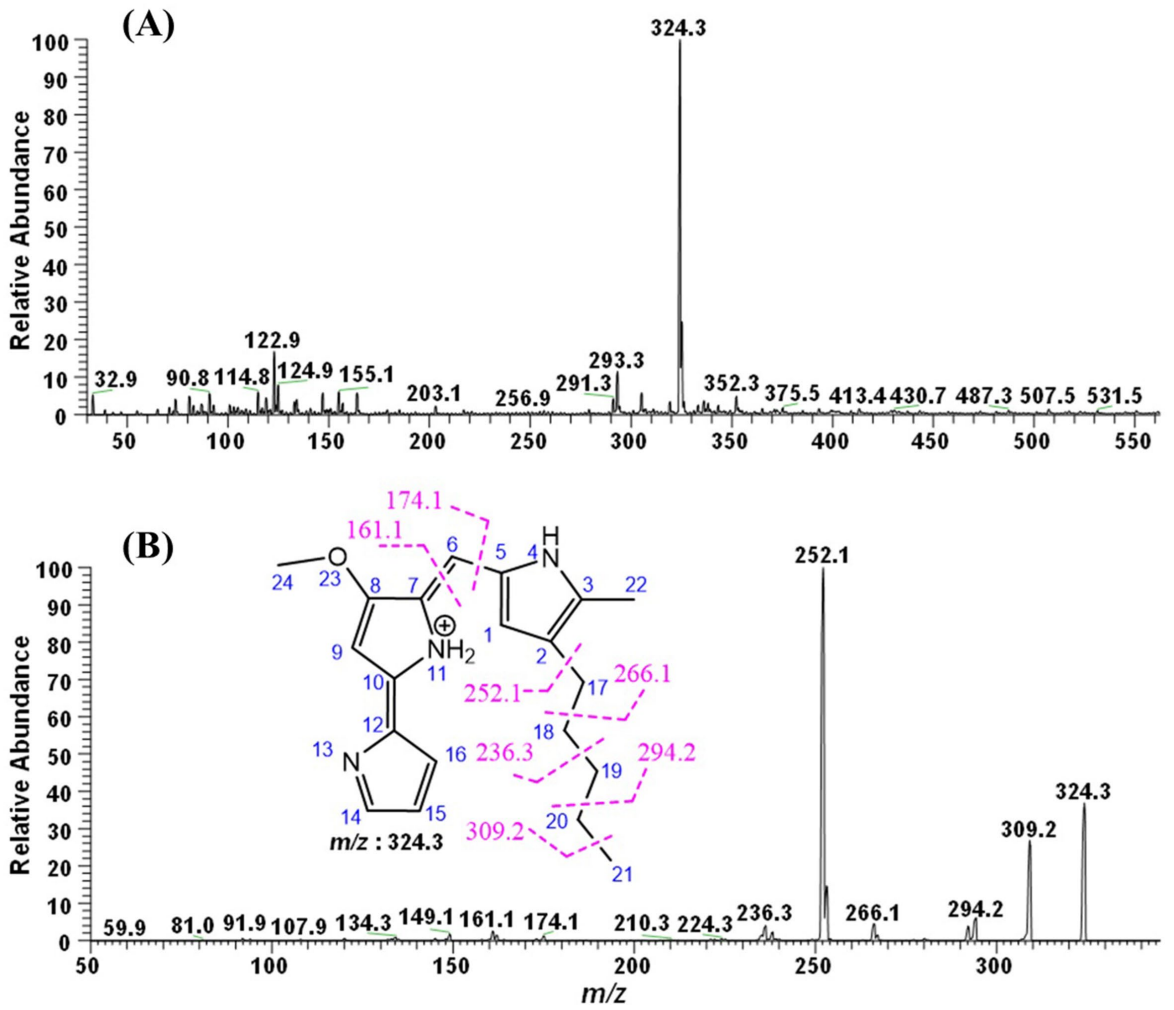
ESI-MS/MS analysis of the pigments. **(A)** Full MS **(B)** ESI-MS2 of m/z 324.3.

In order to determine the molecular weight and characterize the red pigment sample, it was subjected to tandem mass spectrometry using a TSQ Quantis triple quadrupole mass spectrometer (Thermo Electron Corporation, USA), coupled with a direct-injection system. The ionization of the molecular moieties was conducted in the positive mode using an Electron Spray Ionization (ESI) probe. For this analysis, the pigment was dissolved in absolute methanol, and 20 μL was injected into the system. The instrument was operated in product ion scan mode from m/z 30 to 2000. The scan rate was 1,000 Da/s, and the collision energy was varied from 15 to 29 V using argon gas. The CID was kept between 2.9 and 5 m Torr, while the capillary voltage was 3.5 kV and the temperature of the transfer capillary was maintained at 285 °C. The spectral data were characterized manually, and the chemical structures were drawn using ChemDraw Ultra 8.0 software (Figure 1).

### 2.7 Bioactivities of prodigiosin

#### 2.7.1 Antibacterial assay

The antibacterial activity of prodigiosin was evaluated using the agar well-diffusion method against *E. coli* and *B. subtilis* on Muller–Hinton (MH) agar media. The inocula of the selected bacterial strains were prepared in LB broth and incubated for 16 h in a shaking incubator at 37 °C. Next, MH agar plates were inoculated with each normalized bacterial culture of approximately 10^8^ CFU/mL and OD_600_ = 0.1 to obtain a uniform lawn of growth. Subsequently, wells of 5 mm^2^ were formed aseptically in inoculated agar plates with sterile microtips. Each filter-sterilized test compound (100 μL) at different pigment concentrations (100, 250, 500, and 1,000 μg/mL) was applied to the prepared wells on an agar plate. The antibiotic gentamycin (100 μg/mL) was used as a positive control against *E. coli* and *B. subtilis*, whereas compound-solvent methanol was used as a negative control. Inoculated plates were placed at 4 °C for 2 h to allow pre-diffusion of the pigment. Subsequently, respective plates were incubated for 18–24 h at 37 °C to allow for the formation of inhibition zones. After incubation, the clear zones of inhibition surrounding the wells were measured (mm). The diameters of the inhibition zones of the test compounds were compared with those of the control and blank to determine the relative antibacterial activities of the test compounds. Furthermore, three biological replicates were performed for each bacterial strain.

#### 2.7.2 Antifungal assay

The antifungal activity of prodigiosin was investigated through the agar well-diffusion method against *Fusarium oxysporum and Aspergillus niger* on Potato Dextrose Agar (PDA) media. The inoculum of the selected fungal strains was prepared in normal saline. Next, PDA agar plates were inoculated with each fungal inoculum to obtain a uniform lawn of growth. Subsequently, wells of 5 mm^2^ were formed aseptically in inoculated agar plates with sterile microtips. A total of 100 μL of each filter-sterilized test compound at different pigment concentrations (100, 250, 500, and 1,000 μg/mL) was applied to the prepared wells on an agar plate. The antifungal compound nystatin (100,000 units/mL) was used as a positive control against the fungal strains, whereas methanol (solvent) was used as a negative control. Inoculated plates were placed at 4 °C for 2 h, allowing the pre-diffusion of pigment. The respective plates were incubated at 30 °C for 72 h to allow the formation of inhibition zones. After incubation, clear zones of inhibition surrounding the wells were measured (in mm). The diameters of the inhibition zones of the compounds were compared with those of the control and blank to determine the relative antifungal activity of the test compounds. Furthermore, three biological replicates were used for each fungal strain.

#### 2.7.3 Antioxidant assay

Antioxidant activity of prodigiosin was evaluated using the 2,2-diphenyl-1-picrylhydrazyl (DPPH) free radical scavenging assay. Solutions of pigment concentration (100, 500, and 1,000 μg/mL) were prepared in absolute ethanol. A DPPH solution of concentration (10 mg/L) was prepared in absolute ethanol. The prepared DPPH solution was stored in the dark for 2 h prior to the assay to stabilize its absorbance. The ideal absorbance of the working solution was adjusted to approximately 0.8–1.0. The reaction was set up in 96-well plates with a total reaction mixture volume (100 μL) per well. Next, 10 μL of each pigment solution and 90 μL of the prepared DPPH solution were added to the respective wells. For the control reaction mixtures, 10 μL of ethanol was added to 90 μL of the prepared DPPH solution. Similar concentrations (100, 500, and 1,000 μg/mL) of ascorbic acid were used as the standards. The prepared 96-well plates were incubated in the dark for 30 min. The absorbance of the reaction mixtures was recorded at 517 nm using a microplate reader. The radical-scavenging activity of the pigments was calculated according to the following equation:


$$\text{Radical Scavenging Activity}\;(\mathrm{RSA})\%=(A_{c}-A_{s}/A_{c})\;x\;100$$

### 2.8 Molecular docking for the binding mechanism

#### 2.8.1 Ligand preparation

ChemDraw 2D was used to draw the chemical structure of the potent molecule, which was identified through experimental research. The structure was stored in the MDL Mol format and subsequently converted into PDB format using Open Babel (O'Boyle et al., 2011). The pre-optimization process was conducted using the MM2 force field [reference] and Chem3D Pro software (Halgren, 1996). The compound was subsequently converted to PDBQT format by uploading it to PyRx 0.8 (Dallakyan and Olson, 2014). In the interim, in preparation for structure-based virtual screening, the target receptors (3il9), (7yuy), and (2e1q) were added to the PyRx AutoDock panel (Trott and Olson, 2010).

#### 2.8.2 Molecular docking analysis

The two-dimensional structures of the compounds were constructed and optimized using the ChemOffice 2012 software suite (Li et al., 2004). A single unit of the FabH enzyme was selected, assigned Gasteiger charges, and subjected to 1,500 minimization steps, comprising 750 steps of conjugate gradient and 750 steps of steepest descent, using the UCSF Chimera (Pettersen et al., 2004). The molecular docking of the compounds with FabH, a conserved broad-spectrum target found in both *E. coli* and *B. subtilis* (PDB ID: 3il9), was performed using the PyRx software in conjunction with the Autodock Vina plugin (Trott and Olson, 2010). A structure-based molecular docking was used to facilitate unrestricted ligand binding to the receptor. The center of the search space was defined at certain XYZ coordinates for each of the three selected targets. Herein, the grid centers were established as follows: 9.90 Å, 62.47 Å, and 51.26 Å for FabH; 110.60 Å, 17.72 Å, 17.15 Å, and 5.26 Å for FKS1; and 101.18 Å and 113.83 Å for Keap1. The dimensions of the grid box were specifically designed to accommodate X = 41.86 Å, Y = 33.42 Å, and Z = 50.23 Å for the ACE2 complex with PDB ID 3IL9. The grid dimensions of the ACE2–2FLU complex were measured at 76.40 Å, 70.38 Å, and 71.80 Å. Similarly, the grid box for the ACE2–7YUY complex was defined with the following parameters: X = 32.84 Å, Y = 38.83 Å, and Z = 41.46 Å.

During the docking process, the coordinates of the Asn247: OD1 atom were established as centres. For each molecule, 10 interactions were generated within a 10 Å radius of Asn247: OD1. The docking affinity of the compound to the protein was assessed using the binding affinity score. In AutoDock Vina, the chemical affinity of the protein was assessed in terms of the binding energy. The UCSF Chimera and Discovery Studio were used to analyze the binding conformation and interactions of drugs within the active site of the protein (Biovia, 2017). The compound used in the molecular docking process was a pigmented molecule extracted during the experimental approach, specifically characterized by the formula (2E,5Z)-4-methoxy-5-((5-methyl-4-pentyl-1H-pyrrol-2-yl)methylene)-1,5-dihydro-[2,2′-bipyrrolylidene]-1-ium_out. Here, we investigated the compound’s binding affinity against other protein targets, as well as its inhibitory effects against antifungal and antioxidant protein targets. The crystal structure of the protein target, which was conserved between *F. oxysporum* and *A. niger* (PDB ID: 7yuy), was investigated for molecular docking activities using a literature-supported active site (Hu et al., 2023). This has been followed by an antioxidant protein target (PDB ID: 2e1q) from Homo-sapiens for the binding mode of the compound prodigiosin with all the steps mentioned earlier (Yamaguchi et al., 2007).

Because of their different functions in bacterial, fungal, and human cellular processes, FabH (*β*-ketoacyl-ACP synthase III), FKS1 (1,3-β-D-glucan synthase), and Keap1 (Kelch-like ECH-associated protein 1) were selected as typical targets. FabH is a desirable target for selective antibacterial medicines against infections such as *Mycobacterium tuberculosis* and *E. coli* because it catalyzes the first condensation step in bacterial fatty acid biosynthesis (FAS-II), a pathway that is absent in humans (Lu et al., 2015). Fungal cell wall integrity depends on the catalytic subunit of the β-1,3-glucan synthase complex, which is encoded by FKS1. Echinocandin antifungal treatment is based on suppression of this subunit, which results in cell lysis (Ha et al., 2006). Through the Keap1–Nrf2–ARE signaling axis, Keap1, a cytoplasmic repressor of the transcription factor Nrf2, controls oxidative stress responses. In cancer, neurodegeneration, and inflammatory diseases, disruption of this interaction improves cellular antioxidant defenses and has therapeutic implications (Xiao et al., 2024). As a whole, these targets provide a variety of clinically meaningful therapeutic intervention methods for both human and microbial systems.

### 2.9 Statistical analysis

All experimental data related to the bioactivity of the pigment were expressed as the mean ± standard deviation (SD) from three independent replicates (*n* = 3). Statistical analysis of the recorded data was performed using IBM SPSS Software (version 27). The statistical significance among different pigment concentrations and bioactivities was evaluated using one-way and two-way analysis of variance (ANOVA), followed by a post-hoc test (Tukey’s HSD) to determine pairwise comparisons of the effects of pigment concentrations. Graphs were generated with error bars indicating standard deviation (SD), and a *p*-value of less than 0.05 was considered statistically significant.

## 3 Results

### 3.1 Recovery of *S. marcescens*

**Figure 2:**
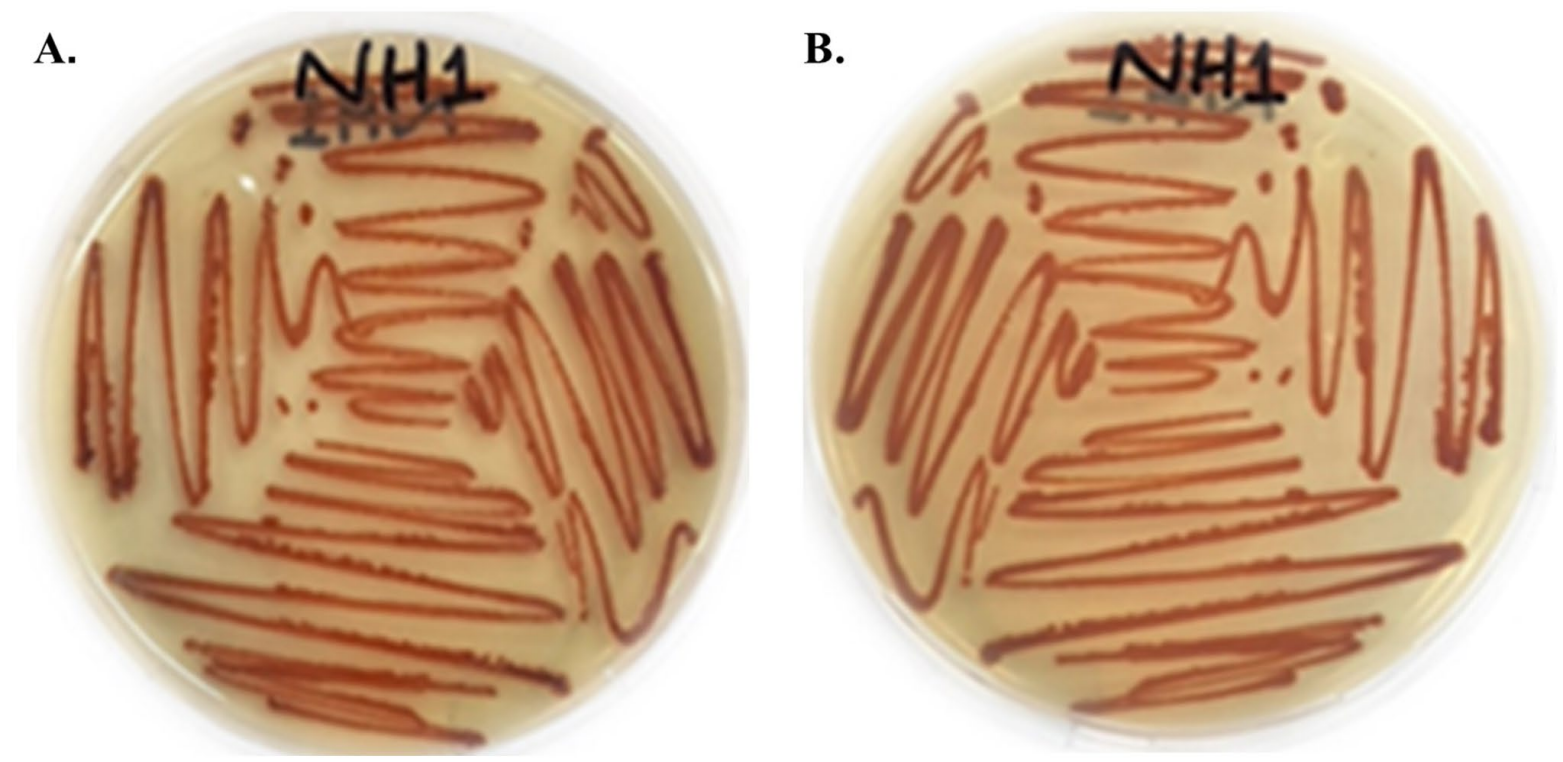
Colony morphology of red-pigmented *S. marcescens* cultured on LB agar after 48 h incubation at 30 °C. **(A)** Front side of culture. **(B)** Back side of culture.

A lawn of colorless, translucent, smooth-edged, convex colonies slightly pigmented in the centre was observed, following an incubation period of 24 h. Facultative anaerobes exhibited optimal growth at 30 °C. After 48 h, vibrant red pigmentation in colonies was observed on the surface of LB agar plates (Figure 2). Subculturing of the producing strains confirmed the viability of the respective strains. Under a microscope, *S. marcescens* was observed as a Gram-negative, rod-shaped, and motile bacterial cell. The appearance of distinct red colonies corresponded with the phenotypic characteristics of *S. marcescens*.

### 3.2 Production of prodigiosin

**Figure 3:**
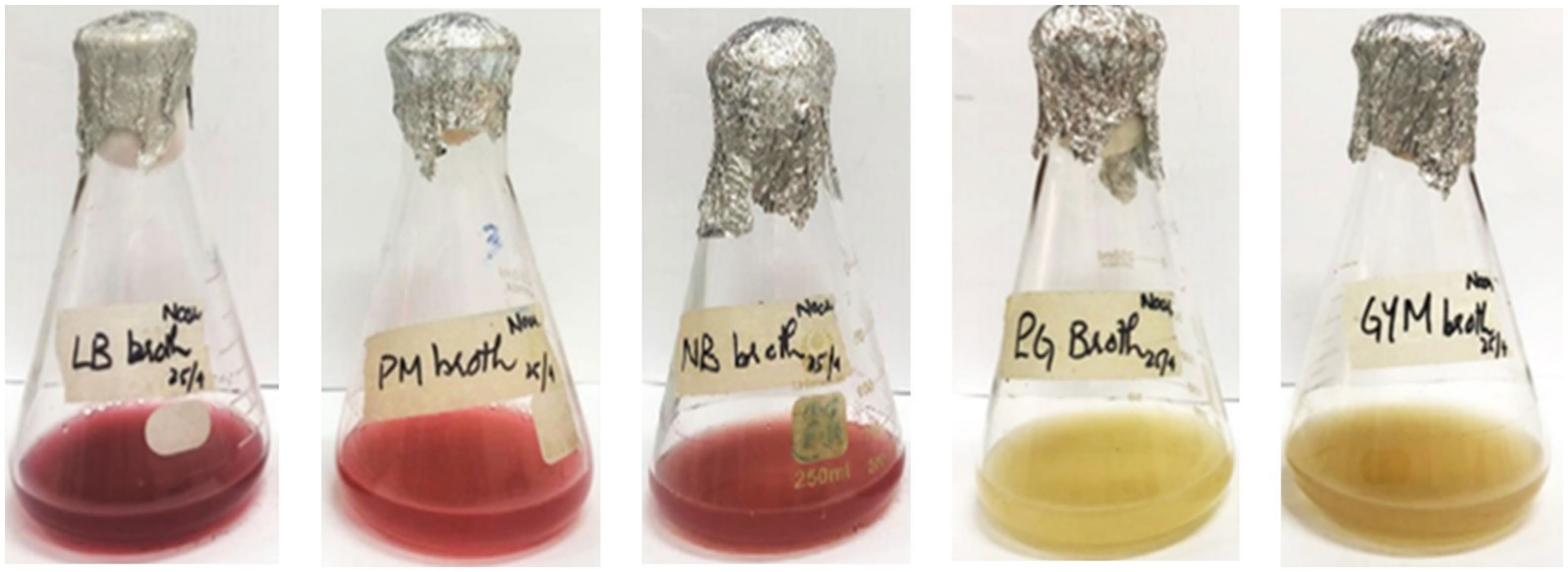
Production of pigment from *S. marcescens* in different production media through submerged fermentation after 72 h of incubation at 30 °C.

Inoculation of seed media with *S. marcescens* resulted in visible growth of bacterial culture after 18–24 h of incubation at 30 °C. Different media, including LB broth, King’s B broth, nutrient broth, PM broth, PG broth, GYM broth, and MM broth, were inoculated with 4% (v/v) inoculum from the seed media. All nutrient media exhibited visible growth of bacterial culture after 18 h of incubation at 30 °C. Among the selected media, PM, LB, and NB broths exhibited visible red pigmentation of *S. marcescens* after 24 h of incubation at 30 °C (Table 1 and Figure 3). However, PM broth exhibited slight pigmentation at 37 °C. The incubation period of the culture was sustained to allow optimal production of pigment. Pigmentation became more pronounced after incubation for 72 h at 30 °C (Figure 3). However, no visible pigment production was evident in the other nutrient media even after 72 h at 30 °C and 37 °C (Table 1). Hence, the PM broth demonstrating the highest efficacy in supporting pigmentation was scaled up to 1,500 mL for the production of red pigment for further analysis.

**Table 1:** Qualitative comparison of bacterial growth and prodigiosin production across different media at incubation temperatures of 30 °C and 37 °C.

Media	30 °C		37 °C	
	Growth	Prodigiosin production	Growth	Prodigiosin production
GYM broth	+	–	+	–
King’s B broth	+	–	+	–
LB broth	+	+	+	–
MM broth	+	–	+	–
Nutrient broth	+	+	+	–
PM broth	+	+	+	+
PG broth	+	–	+	–

‘+’ means growth appearance/prodigiosin production. ‘–’ means no growth/prodigiosin production.

### 3.3 Preliminary identification of prodigiosin

**Figure 4:**
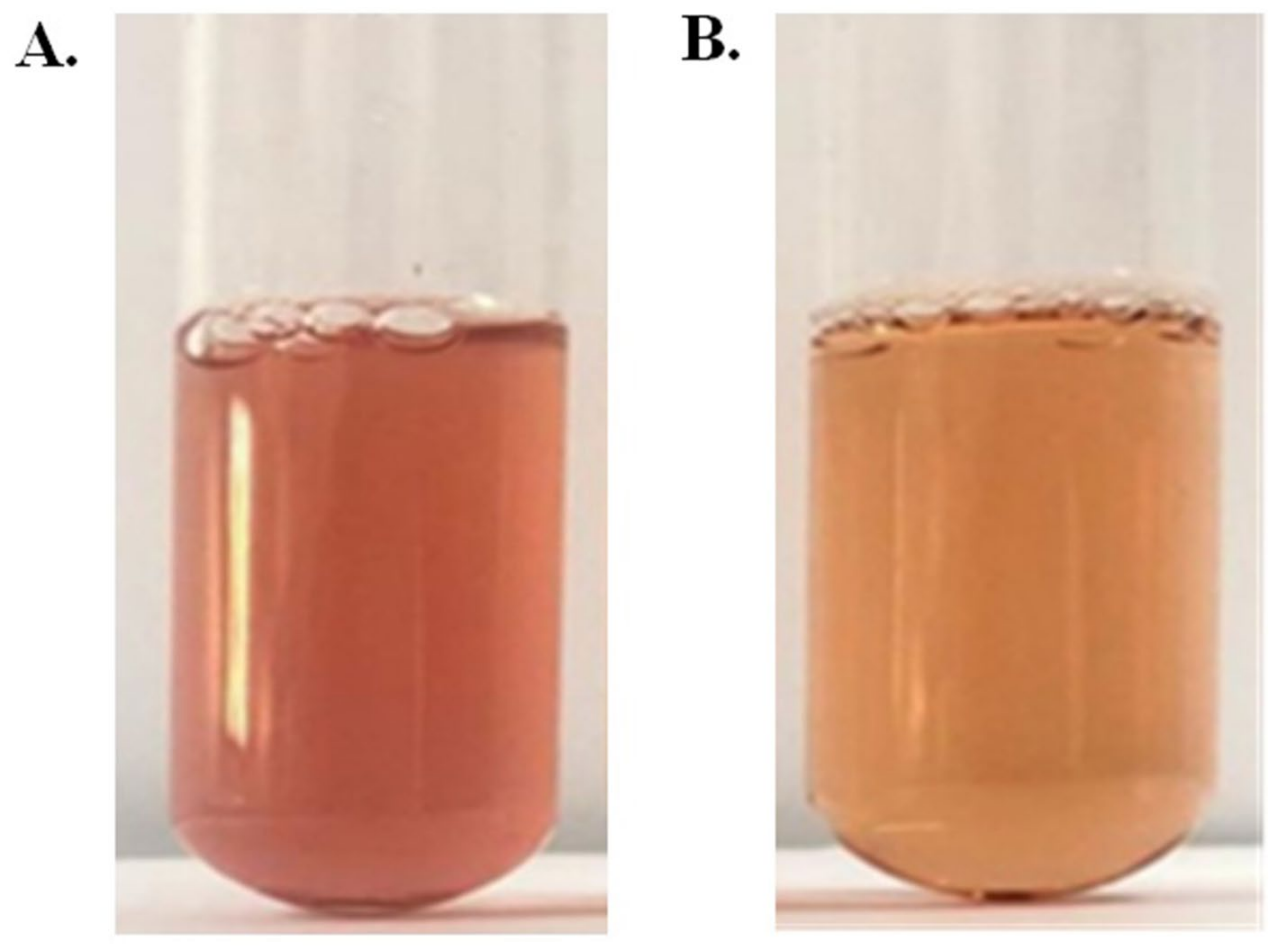
Preliminary identification of pigments. **(A)** Acidified solution. **(B)** Alkalinized solution.

The presumptive color test of prodigiosin was carried out to validate the presence of prodigiosin. The acidified solution of pigment indicated a red or pink color, whereas the alkalinized solution of pigment had a yellow or tan color (Figure 4). This confirmed the presence of a positive presumptive color test for prodigiosin.

### 3.4 Extraction and purification of prodigiosin

**Figure 5:**
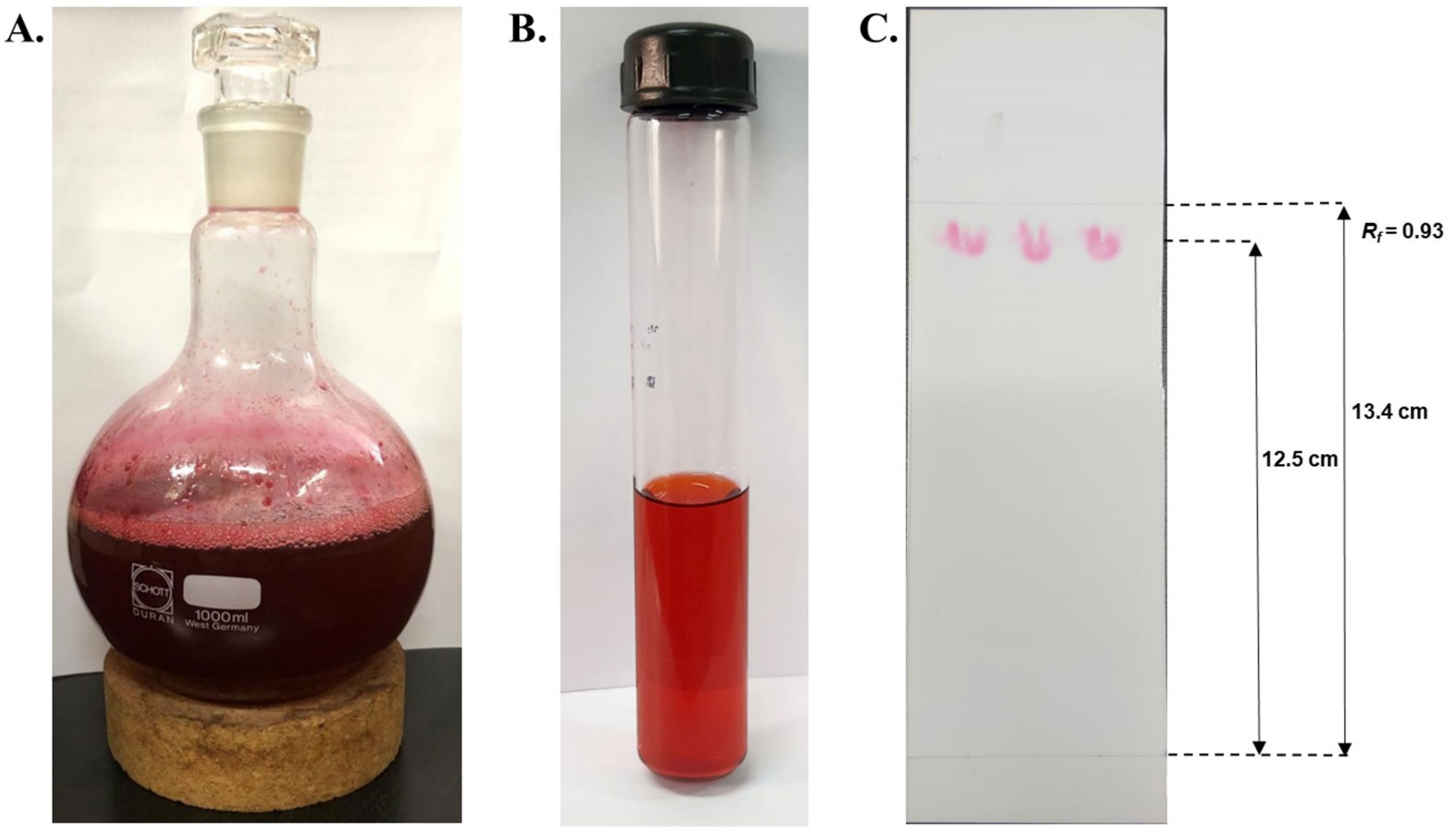
Extraction and purification of red pigment. **(A)** Concentrated crude extract of red pigment extracted using absolute methanol. **(B)** Concentrated eluted fractions of prodigiosin were obtained using silica gel column chromatography. **(C)** TLC plate exhibiting pink spots of prodigiosin with an *R_f_* value of 0.93.

A high solubility of the hydrophobic pigment was observed in absolute methanol, forming a homogenous solution (Figure 5A). The extracted pigment was concentrated using a rotary evaporator to obtain a crude extract of prodigiosin. The red pigment was effectively recovered from the *S. marcescens* cells for further analysis. The extracted crude pigment was subjected to silica gel column chromatography, and the hydrophobic compound, prodigiosin, was eluted using a solvent system of chloroform and methanol (9:1, v/v). The eluted fractions were concentrated for further purification by TLC (Figure 5B). Prodigiosin appeared as a pink spot on the silica gel TLC plate. The calculated *R_f_* value of 0.93 validated the correspondence between the recovered pigment and prodigiosin (Figure 5C).

### 3.5 Characterization of prodigiosin

#### 3.5.1 UV–vis spectrophotometric analysis

**Figure 6:**
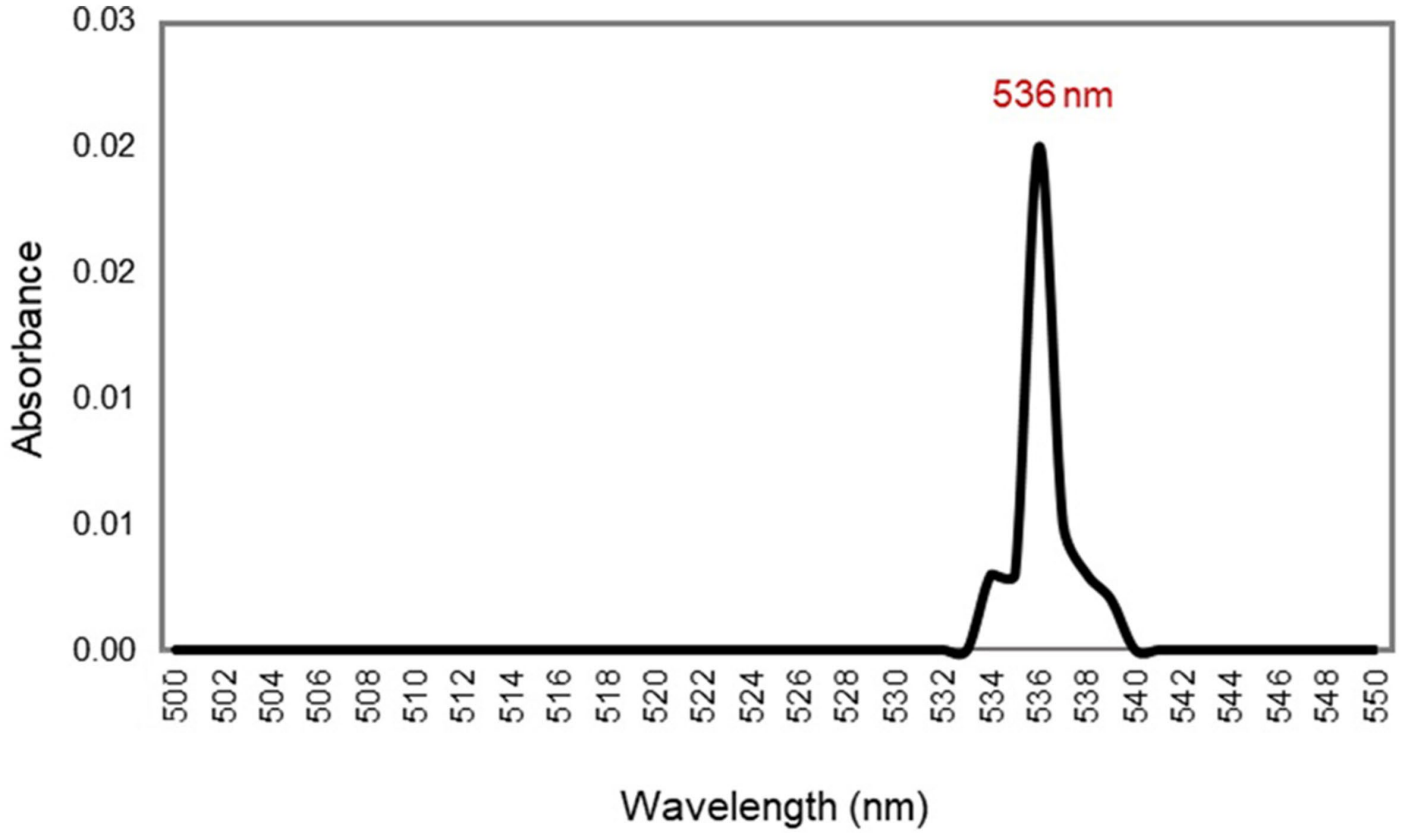
UV–vis spectrum of the red pigment extracted from *S. marcescens,* manifesting λ_max_ at 536 nm.

Intracellular prodigiosin was extracted with absolute methanol, and the absorption spectrum of the purified pigment was recorded. As shown in Figure 6, UV–Vis spectrophotometric analysis of the pigment exhibited a maximum absorbance peak at 536 nm.

#### 3.5.2 Fourier transform infrared (FT-IR) spectroscopic analysis

**Figure 7:**
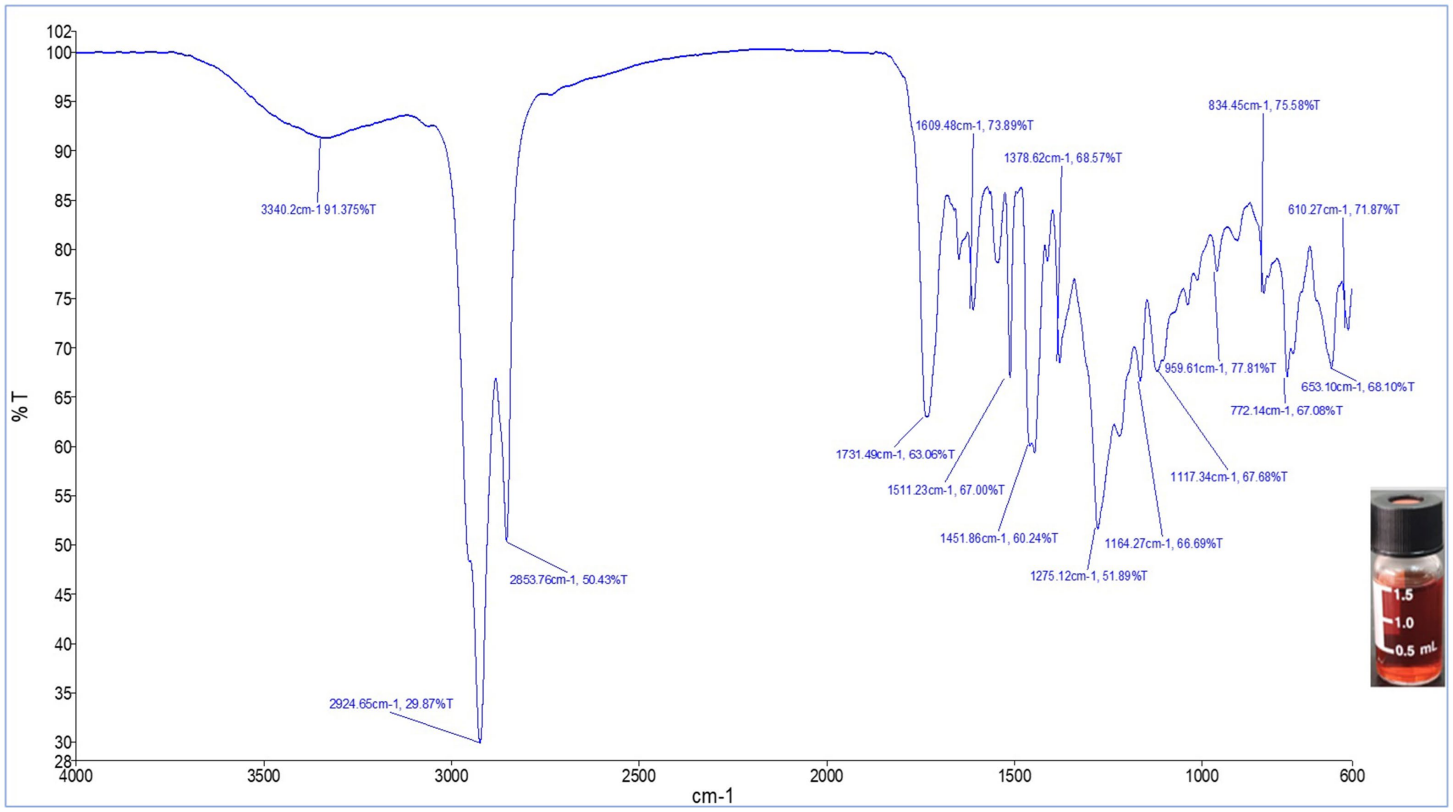
FTIR spectrum of the red pigment extracted from *S. marcescens* in the spectral range 4,000–400 cm^−1^.

FTIR analysis of the TLC-purified pigment was carried out in the spectral range 4,000–400 cm^−1^ for further characterization. As shown in Figure 7, the FTIR spectrum of the red pigment from *S. marcescens* exhibited a broad absorption at 3340.2 cm ^−1^. Strong characteristic peaks were evident at ν_max,_ 2924.6 cm^−1^ and 2853.7 cm^−1^. Whereas, medium-intensity peaks were observed at ν_max_, 1731.4 cm^−1^, 1609.4 cm^−1^ and 1511.23 cm^−1^, 1451.86 cm^−1^, 1378.62 cm^−1^. Furthermore, the fingerprint region of the FTIR spectrum was characterized by peaks at 1275.12 cm^−1^, 1164.27 cm^−1^, 1117.34 cm^−1^, 959.61 cm^−1^, 834.45 cm^-1,^ and 772.14 cm^−1^. The spectrum obtained indicated that the pigment pattern was identical to that of prodigiosin.

#### 3.5.3 ESI-MS/MS of red pigment

During the Full ESI-MS of the red pigment, various positively ionized molecular ion peaks were detected. The distinct ion peaks were m/z 352.3; with base peaks at m/z 324.3, 309.2, 293.3, 155.1, and 122.9. The difference between m/z 352.3 and 324.3 is 28 mass units, which shows the loss of the ethyl group, showing m/z 352.3 as the derivative of the base peak. The m/z 293.3 was obtained because of the loss of 31 mass units (324.3–293.3 = 31), which could be due to the loss of the ethoxy group (Figure 1A).

Further down the line, the base molecular ion peak of m/z 324.3 [M + H]^+^ was isolated and subjected to ESI-MS^2^. The daughter ion peaks obtained during this fragmentation were m/z 309.2, 294.2, and 266.1, and the base peaks were at 252.1, 236.3, 174.1, and 161.1. The ion peak at m/z 309.2 was obtained because of the loss of a methyl group (324.3–309.2 = 15 mass units) either from C-21, which is more labile, or from the C-24 position. The ion peak at m/z 294.2 was obtained because of the fragmentation of the ethyl group at the C-19-20 position. The small peak at 393.3 could be attributed to the loss of the methoxy group due to the cleavage of the C-8 and O-23 bonds. The ion peak at m/z 266.1 and the base peak at m/z 252.1 were due to the fragmentation of the parent molecular moiety at the C-17-18 and C-2-17 positions, respectively. Similarly, the small fragments of m/z 174.1 and 161.1 might be due to the fragmentation at the C-5-6 and C-6-7 positions. The fragmentation obtained during the MS^2^ of red pigment, obtained from the *S. marcescens,* suggests the ion peak at m/z 324.3 to be prodigiosin (C_20_H_25_N_3_O). The remaining ion peaks also correspond to fragments of the protonated parent molecular ion of m/z 324.3 (Figure 1B).

### 3.6 Bioactivities of prodigiosin

#### 3.6.1 Antibacterial assay

**Figure 8:**
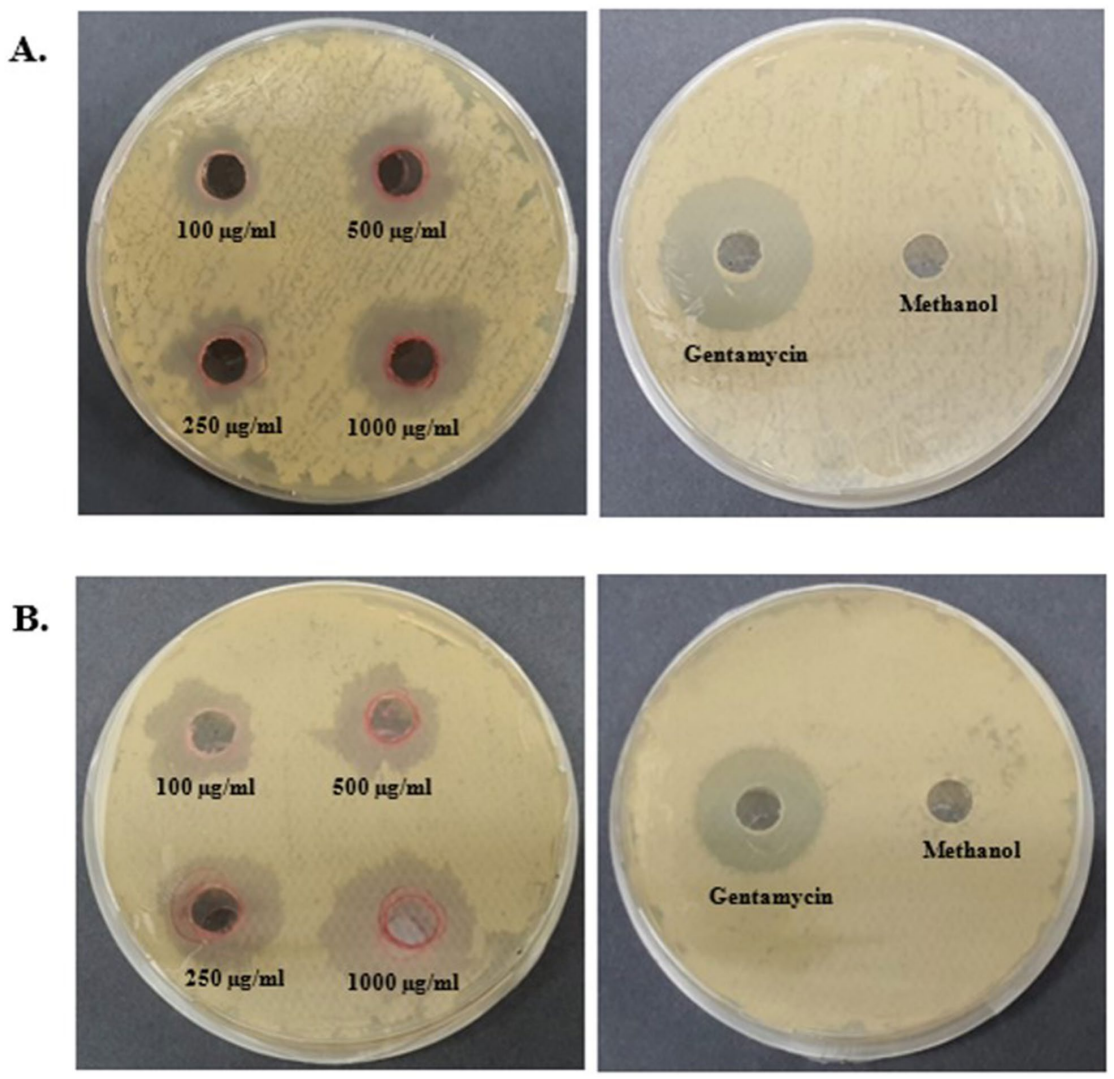
Antibacterial activity of prodigiosin extracted from *S. marcescens*. **(A)** *B. subtilis*. **(B)** *E. coli*.

An antibacterial assay of the pigment was performed using the agar well-diffusion method against *E. coli* and *B. subtilis* at different concentrations (100, 250, 500, and 1,000 μg/mL) (Figure 8). Prodigiosin exhibited an effective inhibitory effect against both Gram-negative and Gram-positive bacteria. The inhibitory effect of the compound increased with increasing concentration of prodigiosin. A Gram-negative bacterial strain of *E. coli* was found to be more susceptible to prodigiosin as compared to the Gram-positive bacterial strain of *B. subtilis*. Prodigiosin exhibited maximum activity against *E. coli* (28.2 ± 0.57 mm) at a concentration of 1,000 μg/mL as compared to *B. subtilis* (23.58 ± 0.6 mm). Minimum antibacterial activity was observed for *B. subtilis* (16.80 ± 0.42 mm) and *E. coli* (19.05 ± 0.78 mm) at a concentration of 100 μg/mL (Figure 8). The antibacterial activity of prodigiosin in comparison to that of the negative control against the tested strains was found to be statistically significant (*p* **<** 0.001). The standard antibiotic gentamycin (100 μg/mL) showed clear zones of inhibition for *E. coli* (24.8 ± 0.42 mm) and *B. subtilis* (27.6 ± 0.57 mm).

#### 3.6.2 Antifungal assay

**Figure 9:**
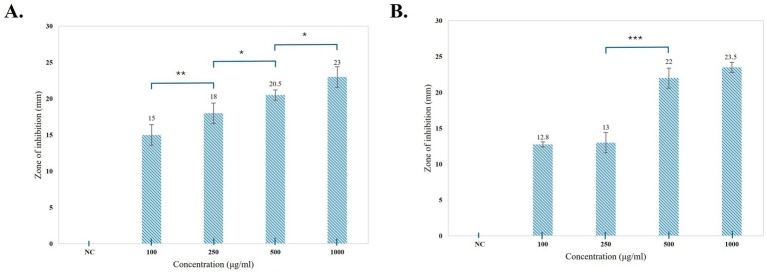
Graphical representation of the antibacterial activity of prodigiosin against the tested strains. **(A)** *B. subtilis*. **(B)** *E. coli*. Bars represent the mean zone of inhibition (mm ± SD, *n* = 3) for each concentration of pigment and negative control. One-way ANOVA demonstrated a significant difference in the activity of pigment at varying concentrations as compared to the negative control (*p* < 0.001). Significance levels were obtained from *post-hoc* Tukey’s HSD for the antibacterial activity of different pigment concentrations, denoted by asterisks: *p < 0.05, **p < 0.01, and ***p < 0.001.

Antifungal assays of pigments were performed using the agar well-diffusion method against *F. oxysporum* and *A. niger* at different concentrations (100, 250, 500, and 1,000 μg/mL) (Figure 9). Prodigiosin showed an effective inhibitory effect against both the tested strains. The inhibitory effect of the compound increased with the concentration of prodigiosin. Prodigiosin exhibited maximum activity against *A. niger* (23.5 ± 0.71 mm) at the highest concentration of pigment (1,000 μg/mL), as compared to *F. oxysporum* (23 ± 1.41 mm). Minimum antifungal activity was observed against *A. niger* (12.8 ± 0.35 mm) at a concentration of 100 μg/mL. The antifungal activity of prodigiosin in comparison to that of the negative control against the tested strains was found to be statistically significant (*p* ***<*** *0.001*). The control Nilstat (100,000 units/mL) showed clear zones of inhibition for *F. oxysporum* (18.5 ± 1.4 mm) and *A. niger* (22.4 ± 0.57 mm).

#### 3.6.3 Antioxidant assay

**Figure 10:**
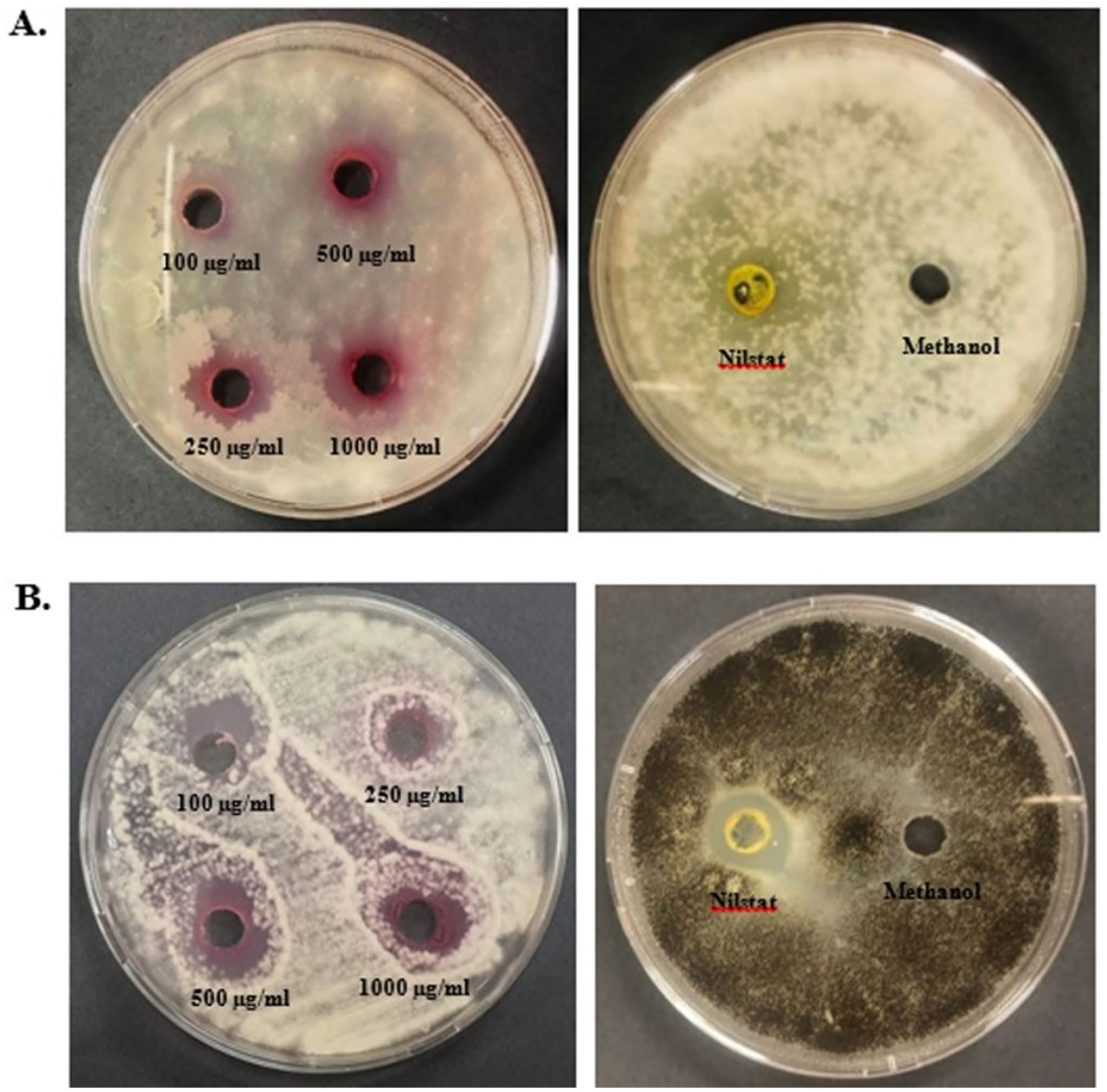
Antifungal activity of prodigiosin extracted from *S. marcescens*. **(A)** *F. oxysporum*. **(B)** *A. niger*.

**Figure 11:**
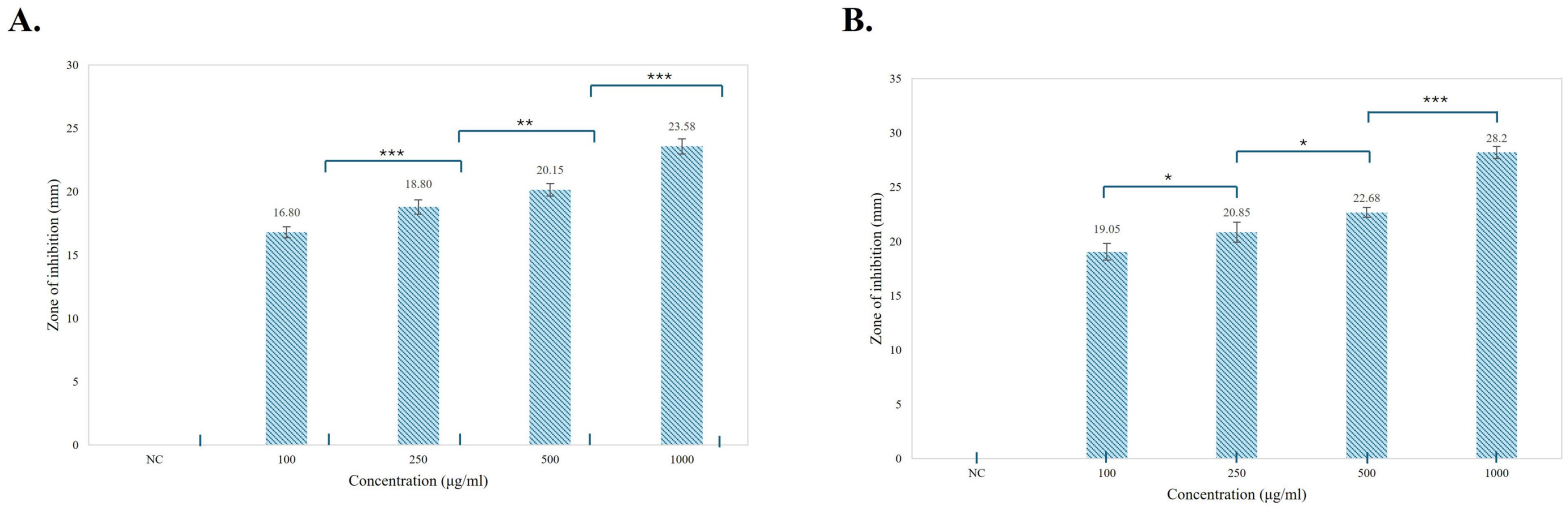
Graphical representation of the antifungal activity of prodigiosin against the tested strains. **(A)** *F. oxysporum* **(B)** *A. niger*. Bars represent the mean zone of inhibition (mm ± SD, *n* = 3) for each concentration of pigment and negative control. One-way ANOVA demonstrated a significant difference in the activity of pigment at varying concentrations as compared to the negative control (*p < 0.001*). Significance levels were obtained using post-hoc Tukey’s HSD for the antifungal activity of different pigment concentrations, denoted by asterisks: *p < 0.05, **p < 0.01, and ***p < 0.001.

**Figure 12:**
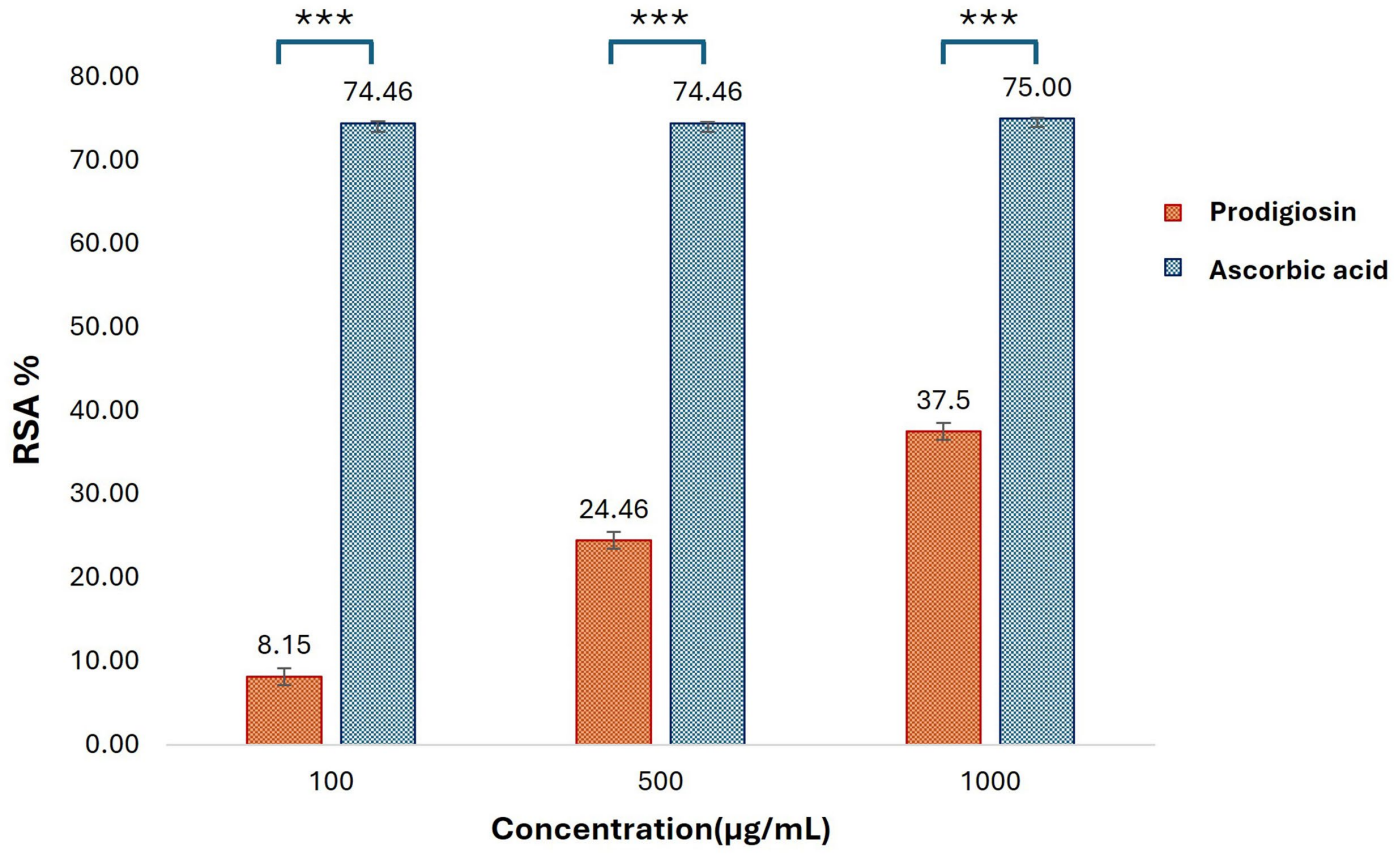
Antioxidant activities of prodigiosin and ascorbic acid at different concentrations were measured using the DPPH assay. Bars represent mean RSA values (% ± SD, *n* = 3) for each concentration of the pigment and ascorbic acid. Statistical significance was determined using two-way ANOVA, presenting a significant effect of group and concentration with a dose-dependent antioxidant response of prodigiosin (*p* < 0.001). *Post-hoc* Tukey’s HSD test was applied for pairwise comparisons (***p < 0.001).

The antioxidant potential of prodigiosin was evaluated using a DPPH free radical scavenging assay. The radical scavenging activity percentage (RSA%) of different concentrations of pigment (100, 500, and 1,000 μg/mL) was calculated. The absorbance of the DPPH reaction mixtures exhibited a significant decline after incubation for 30 min in the dark. Prodigiosin demonstrated significant quenching ability of DPPH free radicals in a dose-dependent manner (*p <* 0.001) (Figure 10). The compound exhibited free radical scavenging activities of 8.15, 24.46, and 37.5% at concentrations of 100, 500, and 1,000 μg/mL, respectively. In contrast, ascorbic acid, which was used as a control, showed strong DPPH radical scavenging activity (75%) at a concentration of 1,000 μg/mL (Figures 11, 12).

### 3.7 Molecular docking studies of prodigiosin

The ring modification of pyrrole-containing compounds may increase their antibacterial, antifungal, and antioxidant activities. One organic compound with a positive charge is −1-ium. Position 4 of the pyrrole ring has a methoxy group, whereas position 5 of the methylene linker has a 5-methyl-4-pentyl-1H-pyrrol-2-yl substituent. The “1-ium” indicates that one of the pyrrole rings’ nitrogen is positively charged. The compound docking scores for the selected antibacterial, antifungal, and antioxidant protein targets are listed (Table 2). The compound was shown to be the most effective against the enzyme after a comparative analysis of the compounds’ antibacterial activity and docking data.

**Table 2:** Molecular docking of the compound against the target proteins with binding scores.

Compound	Protein target with PDB ID	Binding affinities (kcal/mol)
(2E,5Z)-4-methoxy-5-((5-methyl-4-pentyl-1H-pyrrol-2-yl)methylene)-1,5-dihydro-[2,2′-bipyrrolylidene]-1-ium_out	Transferase _ (3IL9)	−7.3
	FKS1 (β-1,3-glucan synthase_ (7yuy)	−7.2
	Keap1 _ 2FLU	−8.3

**Figure 13:**
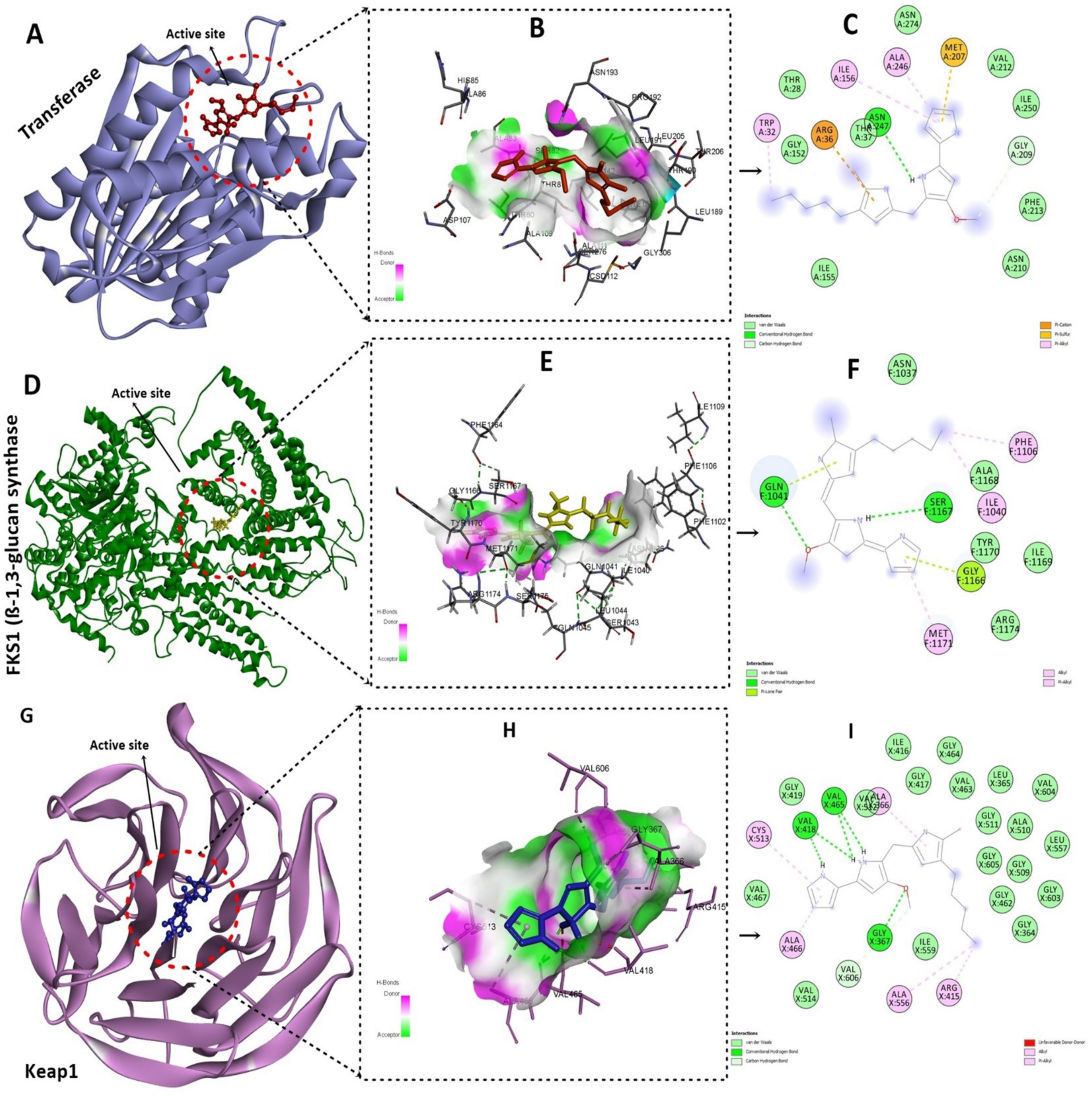
Molecular docking analysis of the compound with target proteins. **(A)** Depicting the FabH protein target with a compound deep inside the binding pocket. **(B)** Presents the hydrogen-bond cloud with H-bond donor and acceptor residues. **(C)** 2D interactions of the compound with FabH protein residues, hydrogen bonds, and other interactions. **(D)** Shows the fungal protein targets along with compounds inside the active site. **(E)** Illustrates the hydrogen bond cloud with hotspot residues with acceptor and donor atoms**. (F)** 2D analysis of the compound with 5-methyl-4-pentyl-2H-pyrrole interacting with protein residues via van der Waals interactions and hydrogen bonding. **(G)** Visualizing the active pocket of the Keap1 human protein target. **(H)** Shows the core residues with hydrogen bond acceptors and donors in the cloud. **(I)** The core pyrrole ring primarily participates in alkyl and pi-alkyl interactions, characterized by strong 5 hydrogen bonds.

The compound inferred a binding energy of −7.3 kcal/mol with a bound orientation and interactions, as shown in Figure 13. One hydrogen bond was found between the anisole ring oxygen atom, the Arg36 residue, and the Phe304 residue. Herein, protein active-site residues participate in hydrophobic interactions in addition to hydrogen bonding, i.e., Asn247, Asn210, Gly209, and Thr37. The compound exhibits distinct binding interactions, presenting deeper binding and distinct binding modes (Figure 13). The chemical compound was oriented along the channel of the active pocket and interacts with a significant portion of the substrate-binding site with Met207, Ala246, Ile156, and Arg36, forming other interactions as well. The pyrrole ring is positioned deep within the cavity, where its oxygen is hydrophilically bonded to the enzyme and shows a high intensity of hydrogen bond donor and acceptor (Figure 13).

During analysis, the docked compound showed a binding affinity of −7.2 kcal/mol against the conserved fungal pathogenic targets of *F. oxysporum* and *A. niger*. The key residues responsible for creating strong hydrogen bonds were Ser1167 and Gln1041, whereas Phe1106, Ala1040, and Gly exhibited alkyl bonds and conventional hydrogen bonds (Figure 13). In addition, the compound had a higher binding affinity of −8.3 kcal/mol with the Keap1 receptor and binds deep inside the cavity (Figure 13). Results indicated a potential hydrogen bond cloud among the target residues and compound atoms, especially with the 5-methyl-4-pentyl-2H-pyrrole moiety (Figure 13). Five hydrogen bonds were found: Val465 and Val418 formed four hydrogen bonds with the nitrogen atoms of the two-ring structure, and one oxygen atom formed a bond with Gly365 (Figure 13), while the other showed van der Waals interactions; the core pyrrole ring mostly participated in alkyl and pi-alkyl interactions.

## 4 Discussion

Microbes and plants are referred to as primary sources of bio-pigments. Microbial pigments offer numerous advantages over plant-based pigments, such as high yield, fast cultivation, cost-effectiveness, sustainability, and industrial-scale production (Nigam and Luke, 2016; Rana et al., 2021). Prodigiosin, violacein, riboflavin, beta-carotene, lycopene, melanin, and phycocyanin have emerged as prominent microbial pigments with multifaceted applications (Sen et al., 2019). However, prodigiosin, a biologically active red pigment well known for its medicinal properties, remains a potential target for scientific research (Herráez et al., 2019). The current study focused on the production, purification, and characterization of a red pigment from *S. marcescens,* followed by the evaluation of the antibacterial, antifungal, and antioxidant activities of the pigment.

The reconstituted strain of *S. marcescens* was inoculated in different production media, including LB, King’s B, Nutrient, PM, PG, GYM, and MM broths. Among these media varieties, PM, NB, and LB broths exhibited visible red pigmentation of *S. marcescens* after 24 h of incubation at 30 °C. PM broth promoted the growth of microorganisms as well as the maximum production of red pigment in a controlled environment. To the best of our knowledge, PM broth, a basic medium comprising peptone and meat extract exclusively, has not been employed for prodigiosin production before. In contrast, multiple studies have explored peptone and meat extract individually as the best nitrogen sources for the maximum production of prodigiosin (Gulani et al., 2012; Hardjito et al., 2002; Lin et al., 2019; Liu et al., 2021; Maurya et al., 2023). In this study, PM broth served as the optimal nutrient source for metabolism and prodigiosin biosynthesis in *S. marcescens*. Peptone is a water-soluble extract of proteins, containing high levels of amino acids, peptides, and long-chain fatty acids (Balasubramaniam et al., 2019). These constituents may provide precursors such as serine and proline for the biosynthesis of prodigiosin (Williamson et al., 2006). In addition, meat extract provides additional carbohydrates, vitamins, minerals, and organic nitrogen sources for the development of microbes.

In accordance with previous studies, NB broth exhibited red pigmentation in *S. marcescens* at 30 °C (Hardjito et al., 2002; Balasubramaniam et al., 2019). Comparatively, the lower pigment production in the Nutrient broth may be attributed to the inhibitory effect of the high concentration of NaCl in the medium, as described in previous studies (Gulani et al., 2012). NaCl causes inactivation of the condensing enzyme required for the terminal step of prodigiosin synthesis (Silverman and Munoz, 1973). LB broth supports the production of pigments but may require modifications for high levels of prodigiosin production, as evaluated by earlier investigations (Gutiérrez-Román et al., 2012; Wei and Chen, 2005). For instance, a high yield of prodigiosin-like pigments has been reported in LB media upon the complete removal of NaCl (Wei and Chen, 2005). This implies that NaCl had a minimal impact on prodigiosin production in *S. marcescens*, as proposed in a previous study (Suryawanshi et al., 2014). Similarly, the current study demonstrated maximal pigment production in PM broth devoid of NaCl. Altogether, it can be inferred that the nutrient medium composition profoundly influences secondary metabolite production in *S. marcescens*. Exploration of the reported optimal carbon and nitrogen substrates may lead to media formulations, promoting yield, color intensity, and stability of pigment (Suryawanshi et al., 2014).

Furthermore, optimal prodigiosin production was effectively recognized in peptone-glycerol media (Elkenawy et al., 2017; Giri et al., 2004), but PG broth and MM broth exhibited no pigment production in this study. Liu et al. reported a similar observation, *in* which glycerol-containing media produced low levels of prodigiosin. *S. marcescens* exhibited no pigment production in GYM broth, possibly influenced by the concentration of glucose (Liu et al., 2021). This result is in accordance with earlier investigations, which marked glucose as a negative regulator of prodigiosin synthesis due to medium acidification or catabolite repression (Kalivoda et al., 2010; Fender et al., 2012). In contrast to a previous study, King’s B medium exhibited no prodigiosin biosynthesis in our study (Venil et al., 2021).

Nonetheless, none of the other production media, except for PM broth, exhibited pigmentation at an incubation temperature of 37 °C. The complete block of prodigiosin production in *S. marcescens* has been reported at 37 °C, in peptone glycerol broth and nutrient broth (Haddix and Werner, 2000). Pigment production in PM broth was comparatively lower at 37 °C, as compared to incubation at 30 °C. This decline in pigmentation in *S. marcescens* has been attributed to the thermoregulation of prodigiosin biosynthesis at the transcriptional level (Romanowski et al., 2019). Although PM broth was identified as an effective medium for prodigiosin production on the basis of qualitative assessment, precise quantification of prodigiosin yield in the respective media is essential for scalability. Further optimization of fermentation conditions and quantitative estimation of prodigiosin production, specifically in PM broth, may facilitate industrial applications of this pigment.

Additionally, the crude extract of the pigment was purified by silica gel chromatography and thin-layer chromatography using a chloroform and methanol (9:1, v/v) solvent system. The prodigiosin appeared as pink spots on the silica gel plate, exhibiting an *R_f_* value of 0.93. The calculated *R_f_* value was determined to be in the range 0.9–0.95, which is a hallmark of prodigiosin. A similar *R_f_* value was stated in the literature by previous studies (Sudhakar et al., 2022; John et al., 2021). In addition, the purified red pigment was characterized by UV–vis spectrophotometry. Spectrophotometric analysis of the methanol extract of the pigment exhibited a maximum absorbance peak at 536 nm, corresponding to the reported fingerprint of the valuable compound prodigiosin (Song et al., 2006; Ren et al., 2017).

To further validate the presence of prodigiosin, FTIR spectroscopy was used for the characterization of the red pigment. The FTIR spectrum of the red pigment from *S. marcescens* exhibited a broad absorption at 3340.2 cm^−1^, corresponding to aromatic N-H stretching attributed to heterocyclic amines of rings A and B (Hernandez-Velasco et al., 2020). Strong characteristic peaks were evident at ν_max_, 2924.6 cm^−1^ and 2853.7 cm^−1^. Interestingly, analogous peaks representing asymmetrical and symmetrical stretching of methyl and methylene groups in the aliphatic chain of the compound have been previously reported (Lin et al., 2019; Faraag et al., 2017). Whereas, medium-intensity peaks were observed at ν_max_, 1731.4 cm^−1^ and 1609.4 cm^-1,^ attributed to C=O and aromatic C=C stretching (Dos Santos et al., 2021; Hernandez-Velasco et al., 2020). The visible peaks at 1511.23 cm^−1^ and 1451.86 cm^−1^ were comparable with results observed in earlier reports, denoting the presence of aromatic C=C, NO_2_, and C-H stretches in the compound (Suryawanshi et al., 2014). Furthermore, the fingerprint region of pigment was characterized with peaks at 1275.12 cm^−1^, 1164.27 cm^−1^, 1117.34 cm^−1^ due to the presence of ester sulphate, C-N, and C-O groups in the structure, corresponding to reported peaks of the characteristic compound (Mathlom et al., 2018). C=C bending caused the spectra at 959.61 cm^−1^, while the C-H phenyl ring occurred at 834.45 cm^−1^ and 772.14 cm^−1^. A previous study indicated similar signals for the fingerprint region in the reported spectrum (Faraag et al., 2017). Functional groups comparable to those reported in the literature suggest that the purified red pigment, prodigiosin, is a valuable molecule.

The ESI-MS/MS analysis of the red pigment from *S. marcescens* depicted that the molecular ion peak at m/z 324.3 [M + H]^+^ is in accordance with the molecular weight of the prodigiosin ion [C_20_H_25_N_3_O + H]^+^ (Arivuselvam et al., 2023). Taken together, these results indicated that the pigment product obtained in this study was prodigiosin.

The multifunctional compound, prodigiosin, is well known for its remarkable bioactivities. In this study, the antibacterial activity of red pigment produced by *S. marcescens* was evaluated against *E. coli* and *B. subtilis*. Different concentrations of prodigiosin manifested significant antibacterial activity against both Gram-positive and Gram-negative bacteria, with a zone of inhibition ranging from 16.80 to 28.2 mm in diameter. The antibacterial effect of the pigment was evident in a dose-dependent manner, which was in accordance with previous studies (Othman et al., 2019; Sudhakar et al., 2022). A higher concentration of prodigiosin (1,000 μg/mL) exhibited considerable growth inhibition of *E. coli* (28.2 ± 0.57 mm) as compared to *B. subtilis* (23.58 ± 0.6 mm). The prodigiosin compound showed significantly lower activity against the test strains in comparison to the standard antibiotic, gentamycin. However, the results indicate that prodigiosin is more effective against Gram-negative *E. coli* as compared to Gram-positive *B. subtilis* under the stated conditions of the agar-diffusion assay.

Previously, Arivuselvam et al. and Sudhakar et al. also reported higher antibacterial activity of prodigiosin against Gram-negative bacteria as compared to Gram-positive bacteria (Arivuselvam et al., 2023; Sudhakar et al., 2022). This difference may be attributed to the distinct cell wall compositions of Gram-positive and Gram-negative bacteria. In addition, multiple mechanisms have been proposed to elucidate the mode of action of prodigiosin in bacterial cells. It is primarily involved in bacterial DNA cleavage, intracellular pH modulation, and cell cycle inhibition (Yip et al., 2021). The effective bactericidal activity may be associated with the presence of the methoxy group in the prodigiosin molecule. The methoxy group, comprising high electron charge density, interacts with the carbonyl group of the peptide chains in the bacterial cell membrane and causes rupturing of the bacterial cell (Arivizhivendhan et al., 2015).

The red pigment manifested significant antifungal activity in addition to its antibacterial activity. The antifungal effect of pigment against *F. oxysporum* and *A. niger* demonstrated a zone of inhibition ranging from 12.8 to 23.5 mm in diameter. In particular, a higher concentration of prodigiosin (1,000 μg/mL) exhibited a greater zone of inhibition (23.5 ± 0.71 mm) for *A. niger* as compared to *F. oxysporum*. In previous studies, prodigiosin has exhibited a significant antifungal effect on *A. niger* and *F. oxysporum* (Suryawanshi et al., 2014). The antifungal potential of prodigiosin is primarily associated with the specific cell wall composition of fungal strains in prior studies (Duzhak et al., 2012). The antifungal potential of prodigiosin makes it an effective biocontrol agent for phytopathogenic fungal strains.

Furthermore, the antioxidant activity of prodigiosin was evaluated using the DPPH radical scavenging assay. The antioxidant activity of prodigiosin increased gradually with higher concentrations of pigment. A similar trend was reported in a previous study on *S. marcescens* (Arivizhivendhan et al., 2018). The highest radical scavenging activity of prodigiosin (37.5%) was observed at a concentration of 1,000 μg/mL, which was comparatively lower than that of the scavenging activity of standard ascorbic acid at the same concentration. These results are supported by previous reports on the elaboration of the antioxidant activity of prodigiosin (Sudhakar et al., 2022; Othman et al., 2019). This infers that prodigiosin has considerable antioxidant potential, which is relatively lower in comparison to strong antioxidants, such as ascorbic acid, and may require further optimization or combination with other bioactive molecules for enhancing the antioxidant efficacy of the pigment. Evidently, antioxidants decrease the absorbance of DPPH reaction mixtures due to the donation of protons to free radicals. This neutralization of charges and loss of free radical properties are marked by a change in the characteristic color of the DPPH solution from violet to light yellow. Decoloration of the DPPH reaction mixture increases with higher concentrations of antioxidant compounds (Nanjo et al., 1996).

Finally, a molecular docking approach was used to evaluate the efficacy of prodigiosin as a potential therapeutic agent by determining its binding affinity towards target drug sites. The pigment showed good affinity towards the target FabH of both *E. coli* and *B. subtilis.* According to earlier research, pyrrole ring derivatives were revealed to be strong inhibitors of *E. coli* and *B. subtilis* FabH enzymes; as a result, they were taken into consideration as receptors in the docking assessment (Lv et al., 2009). The published *E. coli* FabH co-crystallized ligand structure (PDB ID: 3il9) was used to determine substrate-binding site information. Because both enzyme monomers shared the same active pocket, only one was used for docking. In addition, prodigiosin was found to be a good candidate because of its affinity for binding to the target site FKS1 in *F. oxysporum* and *A. niger.* To our knowledge, the binding abilities of red pigment with potential target sites within *F. oxysporum* and *A. niger* were evaluated for the first time in the current study. Furthermore, a molecular docking technique was used to elucidate the potential binding mechanism and affinity of prodigiosin for the antioxidant targets. As per previous results, prodigiosin showed a higher binding affinity for the Keap1 receptor and was bound deep inside the cavity. According to previous reports, compounds that interact with Keap1 alter its conformation, causing Nrf2 to dissociate from Keap1 and move into the nucleus, where it binds to ARE to lessen oxidative stress (Li et al., 2019).

## 5 Conclusion

The current study focused on the production and evaluation of the bioactive potential of the red pigment from *S. marcescens*, unfolding the scope of the secondary metabolite as a microbial pigment. Among the multiple tested production media, PM exhibited the highest efficacy in supporting red pigment production, elucidated as prodigiosin through UV–visible spectrophotometry, FTIR, and ESI-MS/MS analysis. Nonetheless, prodigiosin, produced from *S. marcescens*, exhibited significant antibacterial, antifungal, and antioxidant activities, with molecular docking studies suggesting promising enzyme inhibitory properties. These findings highlight the bioactive potential of prodigiosin in pharmaceutical and agricultural industries, focusing on the growing demand for sustainable and natural bioactive compounds. However, further research is required for the optimization of fermentation conditions, large-scale production of prodigiosin, toxicity and stability assessment, and *in vivo* efficacy evaluation for translation into practical applications and commercial development.

## Data Availability

All relevant data is contained within the article.
